# The Use of Carcasses for the Analysis of Cetacean Population Genetic Structure: A Comparative Study in Two Dolphin Species

**DOI:** 10.1371/journal.pone.0020103

**Published:** 2011-05-31

**Authors:** Kerstin Bilgmann, Luciana M. Möller, Robert G. Harcourt, Catherine M. Kemper, Luciano B. Beheregaray

**Affiliations:** 1 Marine Mammal Research Group, Graduate School of the Environment, Macquarie University, Sydney, New South Wales, Australia; 2 Molecular Ecology Laboratory, Department of Biological Sciences, Macquarie University, Sydney, New South Wales, Australia; 3 School of Biological Sciences, Flinders University, Adelaide, South Australia, Australia; 4 South Australian Museum, Adelaide, South Australia, Australia; Natural History Museum of Denmark, Denmark

## Abstract

Advances in molecular techniques have enabled the study of genetic diversity and population structure in many different contexts. Studies that assess the genetic structure of cetacean populations often use biopsy samples from free-ranging individuals and tissue samples from stranded animals or individuals that became entangled in fishery or aquaculture equipment. This leads to the question of how representative the location of a stranded or entangled animal is with respect to its natural range, and whether similar results would be obtained when comparing carcass samples with samples from free-ranging individuals in studies of population structure. Here we use tissue samples from carcasses of dolphins that stranded or died as a result of bycatch in South Australia to investigate spatial population structure in two species: coastal bottlenose (*Tursiops* sp.) and short-beaked common dolphins (*Delphinus delphis*). We compare these results with those previously obtained from biopsy sampled free-ranging dolphins in the same area to test whether carcass samples yield similar patterns of genetic variability and population structure. Data from dolphin carcasses were gathered using seven microsatellite markers and a fragment of the mitochondrial DNA control region. Analyses based on carcass samples alone failed to detect genetic structure in *Tursiops* sp., a species previously shown to exhibit restricted dispersal and moderate genetic differentiation across a small spatial scale in this region. However, genetic structure was correctly inferred in *D. delphis*, a species previously shown to have reduced genetic structure over a similar geographic area. We propose that in the absence of corroborating data, and when population structure is assessed over relatively small spatial scales, the sole use of carcasses may lead to an underestimate of genetic differentiation. This can lead to a failure in identifying management units for conservation. Therefore, this risk should be carefully assessed when planning population genetic studies of cetaceans.

## Introduction

Tissue samples obtained from stranded cetaceans and from entanglements of fisheries or aquaculture interactions have been used for many genetic studies [1]. These include investigations of taxonomic status (e.g. [2], [3], [4]), genetic diversity (e.g. [5], [6]) population genetic structure (e.g. [7]), dispersal patterns [8], and relatedness of individuals within pods (e.g. [9]). These and many similar studies have transformed our understanding of cetacean molecular ecology and stimulated new and productive lines of research. However, the use of animal carcasses may introduce unforeseen biases.

Cetaceans die or live strand for many reasons, including disease, neonatal death, pollutants, entanglement in fishing and aquaculture gear, boat strikes and intentional killing [10]–[12]. For example, mass mortality events caused by morbillivirus infections have affected many cetacean populations around the world [13]–[15]. Incidental fisheries-related mortality has left more than 25 species of dolphins, porpoises and toothed whales threatened worldwide [16]. Well documented interactions with finfish farms resulting in dolphin mortalities have been reported for several regions, including in Australia and Chile [12], [17], [18]. Perhaps the most significant contributor to fishery induced mortality of dolphins is purse-seine fishing, with millions of dolphins killed by the US eastern tropical Pacific tuna purse-seine fishery between the 1970s and 1990s [19].

Studies that assess genetic variability and population structure using stranded animals or individuals from entanglements are either entirely based on these samples [20], [21], or based on a combination of samples from carcasses and biopsied free-ranging individuals (e.g. [5], [22]–[24]. Genetic samples from cetacean carcasses are usually obtained opportunistically, with no means of predetermining sample size or study area, and with an uncertainty of the individual's origin (e.g. drifting may have occurred after death due to ocean currents or wind). In contrast, biopsy sampling free-ranging cetaceans allows samples to be collected selectively with any one of the various sampling techniques which are now widely used (e.g. [25]–[32]). Biopsy sampling has been generally shown to be minimally invasive, producing mild responses of the animals to sampling, with no long term effects observed [25], [27], [32]. The sampling of free-ranging individuals gives more control over the number of individuals sampled in an area, but it also may introduce biases. There may be, for example, unequal sampling probabilities of individuals due to groups associations/ social bonds, due to survey design, or due to avoidance/ non-avoidance of the approaching research vessel. Biases in biopsying may result in over-sampling of closely related individuals leading to a potential overestimate of population structure but this can be corrected posthoc using relatedness analyses.

Assessing population genetic structure on a small to medium spatial scale with samples from cetacean carcasses leads to the question of how representative the location of a stranded animal is with respect to its natural range [33]. If stranded or entangled animals come from an identified population, their origin may be known with certainty [23]. However, in many situations the carcasses may have come from live populations further afield. For this reason, samples from stranded individuals are most useful if they are found relatively soon after death and are more likely to originate from the local region. Whether this is indeed the case, or if carcasses may have drifted, needs to be assessed on a case by case basis since differences in local oceanography (e.g. strong currents or prevailing winds) and species distributions may differ profoundly between regions. Samples obtained from carcasses that died as a result of anthropogenic activities (e.g. bycatch in fishery interactions) are likely to have lived in the area where they were caught, unless the distribution of prey triggered them to move into a new area. There is a higher level of confidence for these samples that the individual's location is within its local range compared to floating or stranded carcasses. In addition, it is important to consider the health/ condition of an individual prior to death because poor health or injuries may affect its movement patterns.

In the Australian state of South Australia (SA), a large number of tissue samples from carcasses of coastal bottlenose (*Tursiops* sp.) and short-beaked common (*Delphinus delphis*) dolphins have been collected over the past two decades by the South Australian Museum (SAM) and associates. These samples were mainly obtained from strandings (ie beach-washed, floating dead, live stranded), fatal entanglements in fishing and finfish aquaculture operations, and illegally killed dolphins [11], [12], [17]. Here we make use of this sample collection of carcasses to assess genetic variability and genetic differentiation of coastal bottlenose and short-beaked common dolphins in South Australia. This is of particular interest since the two species largely differ in their ecology and from earlier studies (based on live sampling), show dissimilar levels of genetic structuring. Genetic analyses of biopsy samples from free-ranging dolphins have previously shown that bottlenose dolphins from Spencer Gulf and western coastal areas in SA belong to two genetically distinct populations [34] and that common dolphins show no genetic differentiation over a similar spatial scale [35]. In the study presented here we test whether analyses of population genetic structure, using only samples from dolphin carcasses, show similar results to our earlier findings based on biopsy sampling live animals. Analyses based on carcass samples alone failed to detect genetic structure in *Tursiops* sp., a species previously shown to exhibit restricted dispersal and moderate genetic differentiation across a small spatial scale in this region.

## Results

### Samples

Our datasets contained carcass samples from 51 bottlenose and 54 common dolphins (Table 1). There was a considerable difference in sample numbers between Spencer Gulf and western coastal regions, which was a result of limited access to dolphin carcasses in the western coast, and the lack of control over where carcasses strand (Table 1). Sequence fragments of 446 bp of the mtDNA control region were used for the analyses. Ten sequences were identified as common bottlenose dolphins and were excluded from the dataset prior to the analyses. No samples were identified as belonging to the Indo-Pacific species. The 51 samples of our dataset were confirmed to represent southern Australian bottlenose dolphins (see [36]). Sequences of common dolphins were compared to sequences of short-beaked and long-beaked common dolphins available in GenBank. All common dolphin carcasses from SA were confirmed to be short-beaked common dolphins.

**Table 1:** Regions in South Australia from which samples of bottlenose and common dolphin carcasses were obtained, with abbreviations, number of samples and genetically identified sex of sampled individuals.

Bottlenose dolphins					Common dolphins				
Region	Abbreviation	Sample size	♀	♂	Region	Abbreviation	Sample size	♀	♂
Western coastal	W coastal	11	3	8	Western coastal	W coastal	11	5	6
Spencer Gulf	SG	40	16	24	Spencer Gulf	SG	43	18	25
									
Total		51	19	32	Total		54	23	31

### Genetic variability

#### Bottlenose dolphins

We detected five haplotypes and six polymorphic sites in the mtDNA control region dataset of SA coastal bottlenose dolphin carcasses (*n* = 51) (biopsied free-ranging bottlenose dolphins, *n* = 84, 10 haplotypes and 9 polymorphic sites; [34]). Amplification of the control region was successful for all but one carcass.

Haplotypic diversity (h) and nucleotide diversity (π) of dolphin carcasses differed little between the two regions (Table 2). The same was found for mtDNA allelic richness (H), corrected for sample size. A comparison of levels of H, for dolphin carcasses from W coastal areas and SG with levels determined for biopsied free-ranging dolphins (same geographic region, see [34]) showed that values for dolphin carcasses were around 20% lower in both regions (W coastal: H_(carcass)_ = 0.59, H_(biopsied)_ = 0.74; Spencer Gulf: H_(carcass)_ = 0,68, H_(biopsied)_ = 0.86).

**Table 2:** Summary of genetic variability of bottlenose dolphins using carcasses collected in South Australia. Values are based on mtDNA control region sequences and six microsatellite loci.

Bottlenose dolphins	Mitochondrial DNA (*n* = 51)					Microsatellites (*n* = 50)				
Region	Sample size	NH	H	h	π	Sample size	NA	AR	H_E_	H_O_
W coastal	11	3	0.6	0.62 (0.10)	0.0050 (0.0033)	11	5.3	3.3	0.70 (0.15)	0.71 (0.18)
SG	40	4	0.7	0.69 (0.03)	0.0046 (0.0029)	39	6.8	3.3	0.69 (0.09)	0.65 (0.16)

NH, number of haplotypes; H, mtDNA allelic richness; h, haplotypic diversity; π, nucleotide diversity; NA, mean number of alleles per locus; AR, microsatellite allelic richness; H_E_, mean expected heterozygosity; H_O_, mean observed heterozygosity. Values in parentheses are standard errors.

For microsatellites we detected a high null allele frequency (21%) at locus KW2, and therefore we excluded this locus from the analysis, consistent with our free-ranging dataset [34]. No significant departures from Hardy-Weinberg equilibrium were observed for the two regions and remaining six loci. There was also no evidence for linkage disequilibrium between all locus pairs. One individual amplified at two loci only and was therefore excluded from the analysis. Allelic richness (AR) was similar for the two regions (Table 2). Observed heterozygosity was higher for W coastal than for SG (Table 2). A comparison of levels of AR for dolphin carcasses with biopsied free-ranging dolphins in the same geographic area [34] showed considerably lower values for dolphin carcasses. AR for carcasses from the W coastal region was found to be almost half the value of that for free-ranging dolphins (AR_(carcass)_ = 3.3, AR_(biopsied)_ = 5.8), and for SG the value was less than half (AR_(carcass)_ = 3.3, AR_(biopsied)_ = 7.9).

#### Common dolphins

For the mtDNA dataset of common dolphins (n = 51), amplification of control region sequences was unsuccessful for three individuals. Datasets of carcasses and free-ranging individuals were compared for the regions W coastal and SG. Sequences of carcasses from W coastal areas and SG yielded 21 haplotypes and 38 polymorphic sites, with relatively high levels of h and π.(Table 3). Values for H were similar for the two regions (Table 3). A comparison of H for dolphin carcasses and biopsied free-ranging dolphins showed that values were also similar (H_(carcass)_ = 0.95, H_(biopsied)_ = 0.93). This is in contrast to the differences we detected for the two types of bottlenose dolphin datasets.

**Table 3:** Summary of genetic variability of common dolphins, inferred from carcasses collected in South Australia. Values are based on mtDNA control region sequences and seven microsatellite loci.

Common dolphins	Mitochondrial DNA (*n* = 51)					Microsatellites (*n* = 52)				
Region	Sample size	NH	H	h	π	Sample size	NA	AR	H_E_	H_O_
W coastal	11	8	0.9	0.93 (0.07)	0.022 (0.012)	11	6.6	5.9	0.70 (0.06)	0.69 (0.15)
SG	40	18	0.9	0.94 (0.02)	0.017 (0.009)	41	11.4	6.1	0.73 (0.11)	0.62 (0.09)

NH, number of haplotypes; H, mtDNA allelic richness; h, haplotypic diversity; π, nucleotide diversity; NA, mean number of alleles per locus; AR, microsatellite allelic richness; H_E_, mean expected heterozygosity; H_O_, mean observed heterozygosity. Values in parentheses are standard errors.

For microsatellites, we found no evidence for the presence of null alleles, and therefore the analysis for common dolphins was based on the original seven loci. Two individuals were excluded from the analysis due to unsuccessful amplification. Significant deviations from Hardy-Weinberg equilibrium were not observed in the regions or for any of the loci. We found no evidence for linkage disequilibrium between any locus pair. AR was similar for the two regions (Table 3). Observed heterozygosity was higher in the W coastal region than in SG (Table 3). Overall AR for common dolphins from W coastal areas (eastern Great Australian Bight) and SG, which represents one genetic population [35], appeared similar between carcasses (AR = 10.9) and biopsied free-ranging individuals (AR = 9.2).

### Genetic differentiation

#### Bottlenose dolphins

For both mtDNA F_ST_ and Φ_ST_, significant differentiation was detected between W coastal areas and SG. Since values for mtDNA F_ST_ and Φ_ST_ were similar, we only report here the value for Φ_ST_ (Φ_ST_ = 0.118, P<0.05). For microsatellites, pairwise comparison of F_ST_ showed highly significant differentiation ( F_ST_ = 0.068, P<0.001). POWSIM suggested a 100% likelihood of estimating F_ST_ correctly when F_ST_ = 0.025 or above, implying that our dataset has sufficient power for accurate F_ST_ estimates.

**Figure 1:**
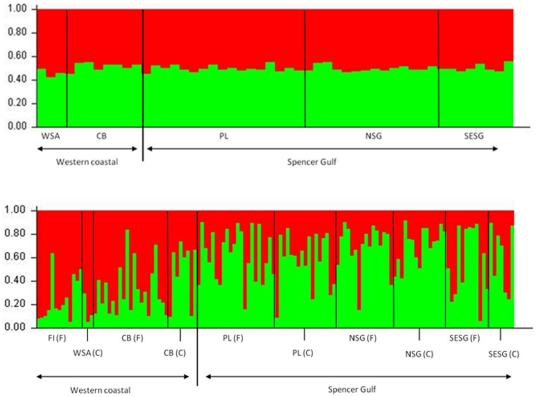
STRUCTURE results for a) bottlenose dolphin carcasses from Spencer Gulf and western coastal areas, South Australia; and b) combined datasets from bottlenose dolphin carcasses and free-ranging individuals, Spencer Gulf and western coastal areas, South Australia. Each vertical column represents one individual dolphin, and the separation of the column into two colours represents the estimated probability of belonging to one or the other population. F = free-ranging dolphins, C = carcasses. Specific geographic locations where dolphins were found or biopsied: FI = St Francis Isles, WSA = western South Australian coast, CB = Coffin Bay, PL = Port Lincoln, NSG = North Spencer Gulf, SESG = southeast Spencer Gulf. See Figure 1 for geographic locations.

STRUCTURE did not detect genetic partitioning for bottlenose dolphin between W coastal areas and SG when using carcass samples, which is in contrast to results based on biopsied free-ranging individuals [34], (Figure 1a). For carcasses, we found the probability of *P*(*X/K*) to be highest at *K* = 1 when using either version of the program (standard version 2.0 and version 2.3.3 for weak population structure) and when applying either the independent or the correlated allele frequency model, suggesting the presence of only one population in W coastal areas and SG of SA when using the carcass dataset. Estimates of *P*(*X/K*) and the prior α showed consistency among multiple runs, indicating that the burn-in length and the length of the runs were appropriate. Additionally, we ran the STRUCTURE analysis by fixing the number of populations to K = 2 and including population location information to identify potential migrants. Three individuals from SG were identified as potential migrants from W coastal. All three carcasses were relatively fresh. One of the three individuals was found near the population boundary to the W coastal area, and the other two individuals were found far away indicating that they were unlikely to have drifted over the population boundary.

A separate STRUCTURE analysis that combined dolphin carcasses and biopsied free-ranging individuals in one dataset led to the identification of genetic differentiation between dolphins from the two areas (Figure 1b). In the majority of runs STRUCTURE suggested the presence of two populations, with a probability of *P*(*X/K*) at *K* = 2 approximating one. The assignment probabilities for free-ranging dolphins to a particular population [34] were on average higher than those for dolphin carcasses (Figure 1b).

**Figure 2:**
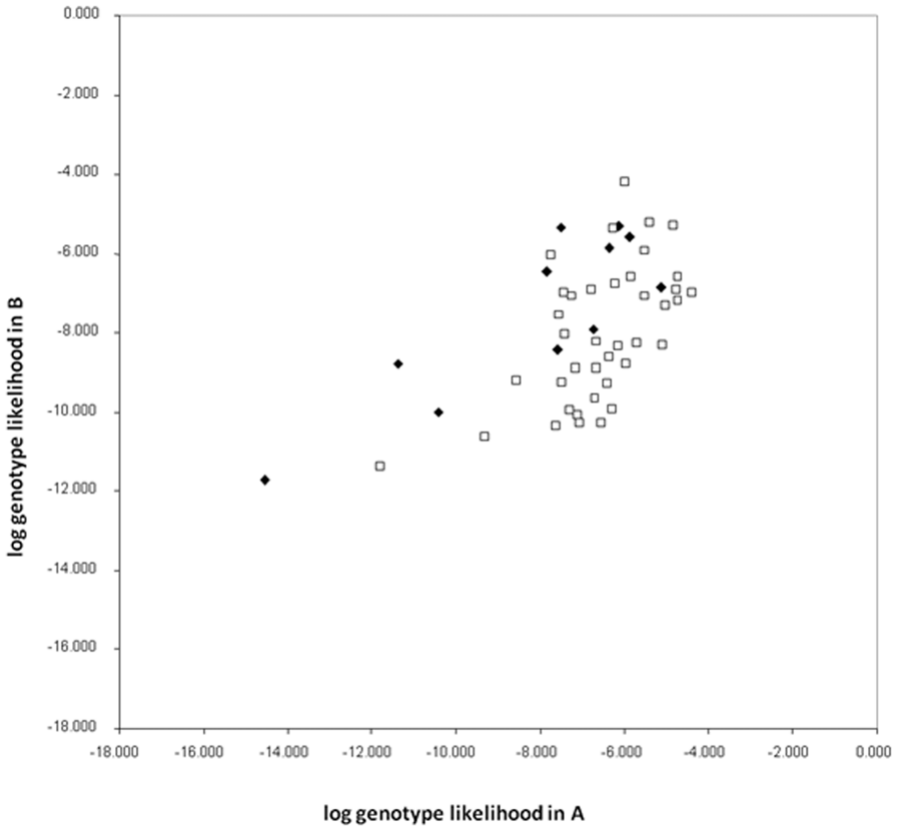
Assignment plot of genotype likelihood values of bottlenose dolphins to population A (Spencer Gulf = black squares) and B (western coastal = white diamonds).

Assignment tests with GENECLASS revealed that although the majority of dolphin carcasses were assigned with higher probability to the area in which they were found, ie W coastal or SG, there was no clear separation of dolphins into these two populations. A large proportion of individuals were assigned to both areas and therefore not rejected from the population in which they showed lower assignment probability. A total of 82.5% of individuals were correctly assigned to their population of origin (based on higher probability), however, the quality index was only 68.52%. As a result of this, the log likelihood plot shows no clustering of dolphins from similar geographic locations into these two areas (Figure 2).

#### Common dolphins

For both mtDNA and microsatellites, we detected no significant genetic differentiation for dolphin carcasses from W coastal areas and SG (Φ_ST_ = 0.059, F_ST_ = −0.014; P>0.05). The test for statistical power of microsatellite F_ST_ values in POWSIM suggested a 99% likelihood of estimating F_ST_ correctly when F_ST_ = 0.01, and a likelihood of 100% when F_ST_ = 0.02 and above. Our carcass dataset therefore has sufficient statistical power to detect relatively low levels of genetic differentiation. Thus the lack of genetic differentiation found for common dolphins from SA is unlikely to be a function of insufficient statistical power.

The lack of genetic structure detected with F_ST_ using microsatellites agrees with results from other analyses, including the Bayesian clustering method implemented in STRUCTURE and assignment tests in GENECLASS (data not shown).

### Genealogical relationships

#### Bottlenose dolphins

**Figure 3:**
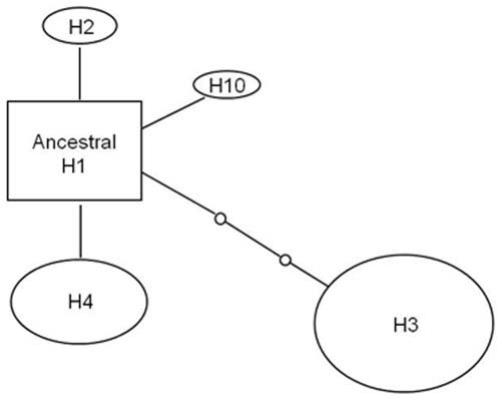
Haplotype network for mtDNA control region sequences of bottlenose dolphin carcasses collected from western coastal areas and Spencer Gulf. The size of the ovals is proportional to the number of individuals showing the particular haplotype. Haplotype H1 was considered to be ancestor based on coalescence theory. Each line indicates one mutation between haplotypes, and small circles between connecting lines represent missing or hypothetical haplotypes.

We constructed a haplotype network using mtDNA control region sequences for bottlenose dolphin carcasses (Figure 3). The network suggests a shallow scenario of matrilineal diversification for bottlenose dolphins in SA, which was also the case for our free-ranging dataset in [34]. H1 had the highest outgroup probability in both networks, representing the most likely ancestral lineage for the area. Most haplotypes appear to have recently originated from this haplotype. The dataset of dolphin carcasses (n = 51) led to the detection of five haplotypes (Figure 3) compared to 10 haplotypes in the free-ranging dataset (n = 84; [34]). All five haplotypes from dolphin carcasses were also present in the free-ranging dataset [34].

**Figure 4:**
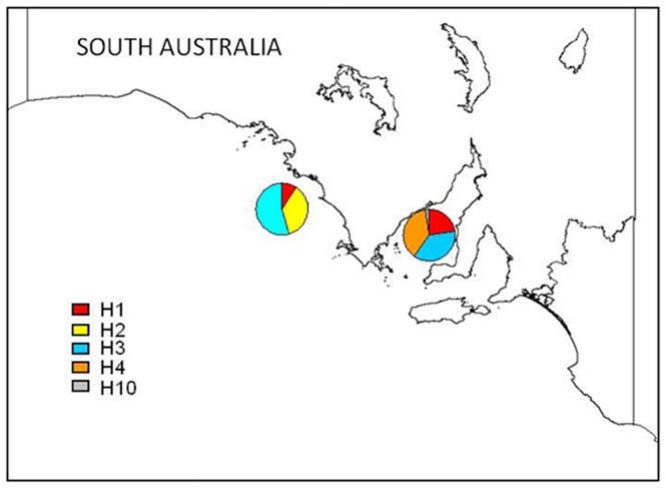
Geographical distribution and frequency of mtDNA haplotypes of bottlenose dolphin carcasses from South Australia.

Distribution and frequency of carcass haplotypes differed between the regions (Figure 4), a pattern we also found for free-ranging bottlenose dolphins [34].

#### Common dolphins

The haplotype network of samples from common dolphin carcasses showed a large number of haplotypes (NH = 21) and a relatively high sequence divergence, with 38 polymorphic sites. The haplotype network is not shown here because it is very similar to that obtained using samples from free-ranging common dolphins (see [35]).

## Discussion

We compared patterns of population genetic structure obtained using carcass samples of South Australian bottlenose and common dolphins with those obtained using samples from biopsied free-ranging individuals in the same geographic area. This comparison is of particular interest because we used two different sample types (carcasses versus biopsy samples) from the same region, and species that differ in their ecology and that were expected to differ in their levels of population genetic structuring. Our study shows that the ability to detect population structure along the South Australian coast was reduced when using samples from bottlenose dolphin carcasses compared to biopsied free-ranging individuals. On the other hand, for common dolphins no genetic differentiation was detected using either dataset.

The previous genetic analysis based on samples from biopsied free-ranging bottlenose dolphins in SA [34] showed that dolphins from Spencer Gulf belong to a different genetic population than those inhabiting coastal waters west of the gulf. Relatedness analyses and estimates of ‘probability of identity’ for biopsied dolphins ruled out that the differentiation detected was due to sampling the same or closely related individuals [34]. In contrast to the biopsy dataset, the carcass dataset for bottlenose dolphins did not show these levels of genetic differentiation. For appropriate management of a species it is critical to correctly detect genetic differentiation. The marine environment often lacks obvious barriers to dispersal [37] and genetic structuring may be higher than expected for species that have the ability to disperse over vast distances [38]. Although marine mammals are generally thought to have a high ability to disperse, some species have been found to exhibit genetic differentiation over small geographic scales (e.g. *Tursiops* sp., [34]; *T. aduncus*, [39]; *Orcinus orca*, [40]; *T. truncatus*, [41]. Restricted dispersal leads to a reduction of the effective size of a population and can therefore lead to a decrease in genetic variability [42]. Thus accurate estimates of genetic differentiation are of great importance in the implementation of management strategies that enable long term population viability.

For South Australian bottlenose dolphins we detected lower levels of mtDNA and nuclear genetic variability in carcass samples. Free-ranging bottlenose dolphins showed levels 20% higher for mtDNA variability in both regions, and for nuclear genetic variability twice the level in Spencer Gulf and more than twice in western coastal areas [34]. Why we detected such low genetic variability for bottlenose dolphin carcasses is unknown. It could relate to individuals with low genetic variability/ high levels of homozygosity resulting in higher susceptibility to diseases [15], however, this remains unknown. Common dolphins appeared similar with respect to genetic variability when both sample types were compared. If the level of genetic variability is reduced for a dataset compared to the actual level in a population, as is the case for SA bottlenose dolphin carcass samples, genetic differentiation may be underestimated, which potentially leads to a failure in correctly identifying management units for conservation purposes. The correct designation of units for management in a species has important implications for the development of conservation and management strategies. Why the common dolphin carcass dataset, in contrast, did not show the same reduced diversity is unknown, but is likely to be due to the different ecology of short-beaked common dolphins and coastal bottlenose dolphins [43]. Free-ranging common dolphins in SA show higher levels of genetic variability [35] than bottlenose dolphins [34].

Genetic studies based on carcass samples may result in other difficulties of interpretation. The location of a stranded individual does not necessarily represent the natural range of the animal [33]. At times, marine mammal carcasses may drift to a location that is outside of the individual's usual range, or diseased and injured individuals may move to shallow waters to rest and die [15]. In addition, when samples from cetacean carcasses are collected over a large time period, such as over many years, it is important to consider whether the area used by individuals may have changed over time. This may be particularly relevant for coastal bottlenose dolphins that may, in some regions, inhabit small home ranges of less than 100 km stretch of coast [44]. To date there is no information available on the size of individual home ranges for bottlenose or common dolphins in SA, therefore it is unknown if this is of relevance to either of the two species in the area.

Our study shows that underestimating population genetic structure can occur when a study is solely based on dolphin carcasses, but does not necessarily apply in all situation and/or species. In many studies samples from dolphin carcasses can be useful and may be the only samples available. However, when planning a study to elucidate population genetic structure that includes samples from dolphin carcasses, in particular when dealing with small to medium spatial scales, we recommend carefully assessing the risk of underestimating genetic differentiation. Our findings may help develop a greater sensitivity towards the use of different types of samples for studies of genetic structure in cetaceans. Here we have shown samples from free-ranging bottlenose dolphins in central SA led to a much more realistic estimate of genetic differentiation, and identification of two Management Units for the area [34], which would have remained undetected in only carcasses were used. This may be because for carcasses (1) genetic variability is potentially lower (2) results can be biased when individuals are found outside of their usual range, and (3) sample sizes are often not ideal due to the unpredictability of stranding events and fatal entanglements, or due to difficulties in accessing remote areas.

### Conclusions

Accurate identification of Management Units may be critical for the conservation of cetaceans, in particular when populations are under threat due to anthropogenic impacts. This is likely to be the case for populations of coastal bottlenose and short-beaked common dolphins in SA, which are impacted by interactions with fisheries and aquaculture for finfish [12], [45], [46], illegal killings [11] and coastal pollution [47].

Our study shows that for bottlenose dolphins in SA but not for common dolphins, samples from dolphin carcasses were not suitable for studies of population genetic structure over small to medium spatial scales, and identification of Management Units in SA was only possible for bottlenose dolphins due to the availability of samples from biopsied free-ranging individuals.

## Materials and Methods

### Ethics Statement

The collection of biopsy samples from free-ranging dolphins was conducted following the protocols in the ethics and research permits. Animal ethics approval was provided by the Department of Environment and Heritage (42/2003) and by the Animal Ethics Committee at Macquarie University (2003/23-2). A research permit (A24720) for the collection of samples from free-ranging dolphins was provided by Department of Environment and Heritage, South Australia.

### Tissue samples

**Figure 5:**
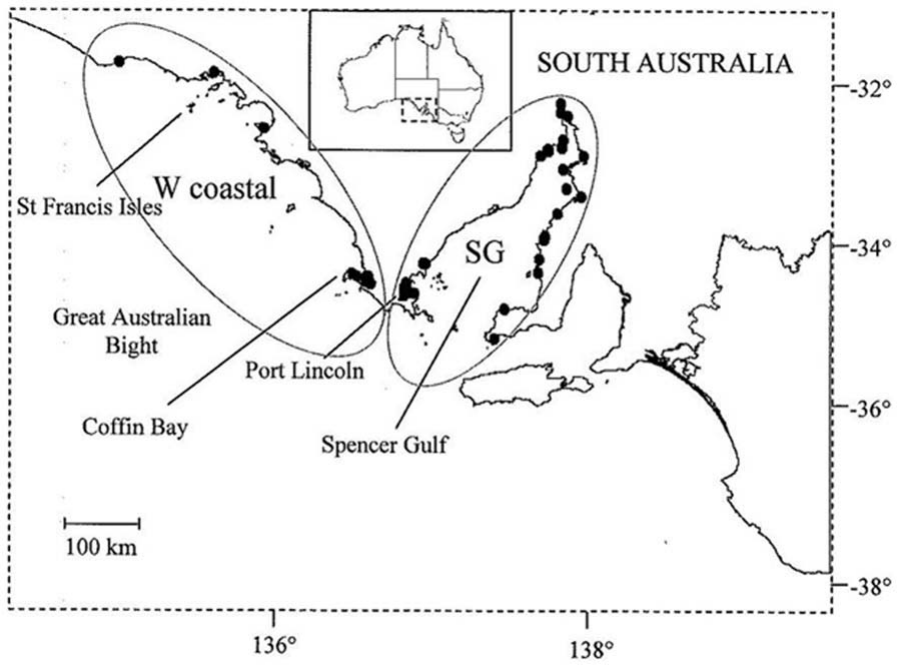
Sampling regions in South Australia from which carcasses of bottlenose dolphins were collected. Black dots within regions represent localities for carcasses. Samples are grouped into two geographic areas: western coastal (W coastal) and Spencer Gulf (SG).

**Figure 6:**
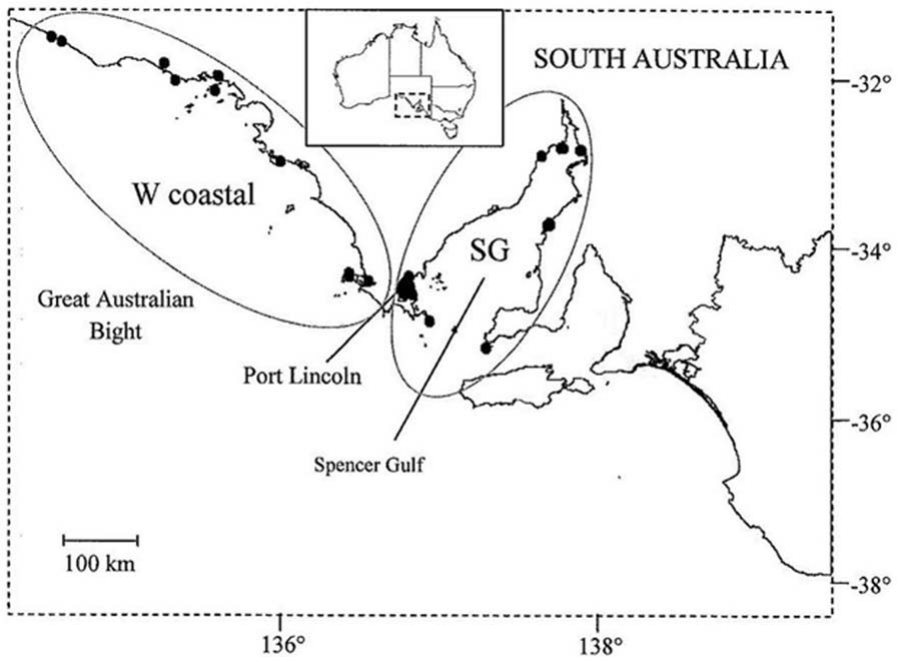
Sampling regions in South Australia from which carcasses of common dolphins were collected. Black dots within regions represent localities for carcasses. Samples are grouped into two geographic areas: western coastal (W coastal) and Spencer Gulf (SG).

Carcasses of stranded coastal bottlenose (*Tursiops* sp.) and short-beaked common (*Delphinus delphis*) dolphins were collected by SAM in the Great Australian Bight and Spencer Gulf (Figures 5 and 6). Carcasses of both dolphin species were also obtained after they became fatally entangled in finfish farm nets in south-western Spencer Gulf (Figures 5 and 6). Dolphin carcasses from stranding events were collected between 1989 and 2003, and from fatal entanglements and other human activities between 1994 and 2000 [11], [17].

As part of the SAM necropsy program, tissue samples have been collected for toxicological and genetic analyses [11]. For this study, we used skin or muscle tissue from relatively fresh carcasses provided by SAM (ie Geraci codes 2–3) [48]. Samples were either frozen or preserved in 70% or 100% ethanol. Each individual recovered was listed in a database created by SAM containing detailed information including the location, date of collection, circumstance of death when known, and decomposition state. For the present study, dolphin samples were grouped into the following regions: western coastal (W coastal) and Spencer Gulf (SG) to match the analysis of our previous study on live sampled individuals (Figures 5 and 6).

### Genetic Methods

We used between 50 mg and 100 mg of subepidermal or muscle tissue for total DNA extraction using a salting out protocol modified from [49]. Individuals were then genetically sexed by amplifying via the polymerase chain reaction (PCR) fragments of the ZFX and SRY genes [50]. PCR conditions were as described in [51].

For each individual a fragment of the mitochondrial DNA (mtDNA) control region was amplified using primers reported in [52], and a reaction mix and PCR conditions described in [53]. Once mtDNA control region fragments were amplified, we screened the fragments by the single-stranded conformation polymorphism (SSCP) method as described in [49]. This technique enables the detection of DNA fragments that are identical in sequence. Multiple samples of each SSCP phenotype and all unique phenotypes were selected for sequencing and sequenced in an ABI 377 automated DNA sequencer following manufacturer's instructions.

We amplified seven polymorphic cetacean microsatellite loci for each individual dolphin: KW2, KW12 [6], EV1, EV37 [54], MK5, MK6 and MK8 [55] using a reaction mix, PCR conditions and gel-electrophoresis methods as described in [51]. The reliability of the PCR was tested by re-amplifying and re-running selected individuals up to three times. In addition, samples with rare and/or large alleles were re-run next to reference samples and a DNA ladder to ensure correct scoring. We used MICRO-CHECKER v.2.2.3 [56] to test for genotyping errors such as non-amplified alleles (null alleles), allele drop-out (i.e. short allele dominance) and stuttering.

In order to confirm that all dolphin carcasses were either from coastal bottlenose dolphins (*Tursiops* sp., see [36]) or short-beaked common dolphins (*Delphinus delphis*), mtDNA control region sequences were compared with sequences from GenBank, and with a large dataset of southern and eastern Australian bottlenose dolphin sequences [36], [53]. This comparison included sequences from Indo-Pacific bottlenose dolphins (*T. aduncus*), common bottlenose dolphins (*T. truncatus*), southern Australian bottlenose dolphins (*Tursiops* sp.), short-beaked common dolphins (*D. delphis*), and long-beaked common dolphins (*D. capensis*).

### Data analysis: mitochondrial DNA

Sequence alignment of mitochondrial DNA (mtDNA) control region sequences was performed in SEQUENCHER 4.1 (Gene Code Corp, USA). Haplotypic and nucleotide diversities were estimated for each species and sampling region using ARLEQUIN v. 2.001 [57]. MtDNA allelic richness was estimated in CONTRIB [58] and compared for the two dataset types (carcasses and biopsied individuals) and geographic regions. Differences between values for mtDNA allelic richness were not tested for significance as this is statistically not possible due to the way they are calculated from the datasets. As a result of this we report percentage differences only. We used ARLEQUIN to assess the degree of genetic differentiation by estimating F_ST_ and Φ_ST_. For the estimates of Φ_ST_, we used the Tamura-Nei genetic distance model [59], determined as the best fit model of sequence evolution for the data by MODELTEST v. 3.06 [59]. Genealogical relationships among mtDNA haplotypes were examined by constructing a haplotype network with TCS v. 1.21 [60], which implements the statistical parsimony method of [61].

### Data analysis: microsatellites

Deviations from Hardy-Weinberg equilibrium and linkage disequilibrium were tested in GENEPOP v. 3.4 [62] using the Fisher's exact test and a Markov chain method with 1,000 iterations. GENEPOP was also used to estimate the mean number of alleles per locus (NA), and the expected (H_E_) and observed (H_O_) heterozygosities for each sampling region. Allelic richness (AR), which takes sample size into account, was determined in FSTAT v. 2.9.3 [63]. We compared AR for dolphin carcasses from Spencer Gulf and western coastal areas with values previously obtained from samples of free-ranging bottlenose and common dolphins from the same geographic area (see [34], [35]. Due to the way AR is calculated from the datasets, it is statistically not possible to test the differences in obtained AR values (AR_(carcass)_ and AR_(biopsied)_) for significance. Comparisons of AR are therefore reported as percentages only, and the results should be interpreted with this in mind.

We estimated genetic differentiation between pairs of sampling regions by F_ST_ using ARLEQUIN [64]. The significance tests were performed using a permutation analysis. The statistical power of F_ST_ estimates for each dataset was tested in POWSIM [65], a program that takes sample size, number of genotyped loci and degree of polymorphism into account to estimate the power to detect true genetic differentiation. To further assess genetic structure in the datasets of dolphin carcasses and to estimate the most likely number of populations (*K*), we used the program STRUCTURE v. 2.0 [66] and the later version to infer weak population structure, v. 2.3.3 [67]. The model used in the later version allows the program to access sampling location information when supportive of ancestry assignment, while ignoring this information from individuals of which ancestry is uncorrelated with sampling location [67]. The new model is designed for datasets with low divergence and thus low statistical power, however, it is recommended to run the original models as well to check if any differences between results from new and old models seem biologically sensible [67]. We used the admixture model and applied both independent and correlated allele frequency models with an initial burn in of 10^5^ iterations and a simulation length of 10^6^ Markov chain Monte Carlo (MCMC) repetitions. For the new model in version 2.3.3., we included information on sampling locations. A series of five independent runs for each value of *K* (1–5) was performed for each dataset to test for consistency in results, convergence of the priors and appropriateness of the burn-in and simulation length. Estimates of the posterior probabilities P(*X/K*) of each run were then assessed to determine the most likely number of *K* for each dataset. The program was applied to a dataset (1) including only carcasses, and (2) including both carcasses and free-ranging individuals, to investigate if the ability to detect population structure increases with combined datasets. Samples solely from free-ranging individuals from SA were analysed prior to this study by employing the same genetic and statistical methods (*Tursiops* sp., [34]; *D. delphis*, [35]).

For the bottlenose dolphin dataset, we further assessed genetic differentiation using assignment tests carried out in GENECLASS v. 2.0 [68]. We use this method to tentatively assign stranded and entangled individuals from Spencer Gulf and coastal areas west of the gulf to two populations previously identified using free-ranging dolphins: (1) Spencer Gulf, and (2) coastal areas west of the gulf [34]. GENECLASS uses multilocus genotypic data to assign individuals to their population of origin, the population for which the multilocus genotype likelihood is highest. Individuals with a likelihood of <5% of belonging to a particular population were considered not assigned to that population. The ‘leave one out procedure’ was applied in order to reduce the bias of assigning the current individual to its source population. We chose a Bayesian method and simulated 10,000 randomly generated genotypes. Type 1 error probability was set to the default value of 0.01 to minimize the risk of erroneous assignment of individuals to their population.
